# Outpatient treatment of varicose veins with endolaser in clinic vs. hospital: cost-benefit and safety assessment

**DOI:** 10.1590/1677-5449.202401602

**Published:** 2025-06-30

**Authors:** Camila Biedler Giordani, Mateus Picada Correa, Luiza Brum Borges, Vitória Cerbaro Farias, Renan Camargo Puton, Jaber Nashat Saleh, Rafael Stevan Noel, Julio Cesar Bajerski

**Affiliations:** 1 Universidade de Passo Fundo – UPF, Passo Fundo, RS, Brasil.; 2 Instituto Vascular de Passo Fundo – Invasc, Passo Fundo, RS, Brasil.

**Keywords:** outpatient surgery, endovenous thermal ablation, varicose veins, cost-benefit

## Abstract

**Background:**

Varicose disease is a common pathology among the population, with an incidence of about 38%. In Brazil, it affects 45% of women and 30% of men. Currently, endovenous thermal ablation (EVTA) of the saphenous vein is the method of choice in the treatment of varicose veins. As a minimally invasive procedure that promotes quick recovery and does not require hospitalization, it can be performed on an outpatient basis.

**Objectives:**

To evaluate the safety and cost-benefit of outpatient varicose vein treatment in a clinic compared to the same procedure performed in a hospital environment.

**Methods:**

A uncontrolled retrospective transversal study of case series evaluated a random group of 50 patients undergoing EVTA for the treatment of varicose disease by the same group of vascular surgeons. Twenty-five underwent the procedure in a tertiary hospital environment and 25 on an outpatient basis. The average costs of room fees and materials from both groups were analyzed and statistically compared with the Student's t-test. Intraoperative and postoperative complications were assessed.

**Results:**

The average hospital cost of the procedure was R$ 1391.99 (± 280.8) in the hospital and R$ 1593.40 (± 99.53) in the clinic. The Student's t-test showed a significant difference with p=0.02. No patient had complications either intraoperatively or postoperatively.

**Conclusions:**

Outpatient surgery, despite having a statistically higher cost than hospital surgery in Brazil, is safe and has a positive cost-benefit for the patient.

## INTRODUCTION

Varicose veins are superficial veins, generally located in the lower limbs, that become twisted and dilated > 3 mm.^1-3^ This condition is quite common, with a worldwide prevalence ranging from 29.5 to 39.0% in women and from 10.4 and 23.0% in men.^2,3^ In Brazil, estimates are similar. According to the *Sociedade Brasileira de Angiologia e de Cirurgia Vascular* (Brazilian Society of Angiology and Vascular Surgery), 38% of Brazilians have varicose veins (45% of women and 30% of men) and, in people aged over 70 years, the prevalence can reach 70%.^4^

Endovenous thermal ablation (ETA) of saphenous veins is progressively replacing conventional surgery as a treatment for saphenous reflux, being strongly recommended in the guidelines.^5^ This procedure is performed percutaneously under tumescent local anesthesia guided by ultrasound.^6^ This allows for a more precise procedure and faster recovery, reducing postoperative hospitalization time and allowing the procedure to be performed on an outpatient basis.^6^

ETA has better results and a lower complication rate than traditional surgery,^3,6^ mainly due to the quick recovery, less pain, and an early return to daily activities.^7^ Although the cost-benefit of outpatient ETA is positive in Europe, it is still unclear in Brazil.^8-10^ Therefore, this study was a safety and cost-benefit assessment of varicose vein treatment in outpatient and hospital settings in Brazil.

## METHODOLOGY

This cross-sectional, retrospective, uncontrolled case series study included 50 patients who were randomly selected based on their medical records by a person who did not otherwise participate in the study. All patients had undergone ETA of saphenous veins and phlebectomy of tributaries by a team with 15 years’ experience in treating venous insufficiency.

**Figure 1:**
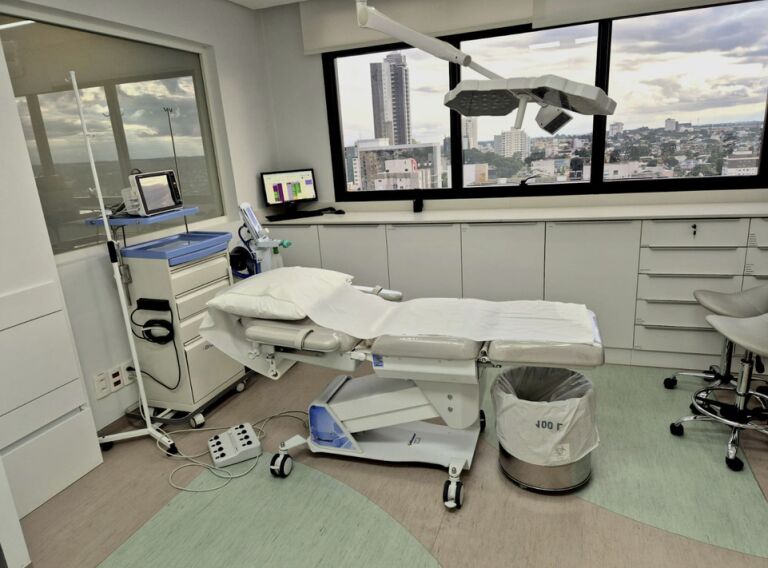
Private outpatient clinic in which the surgeries were performed.

**Figure 2:**
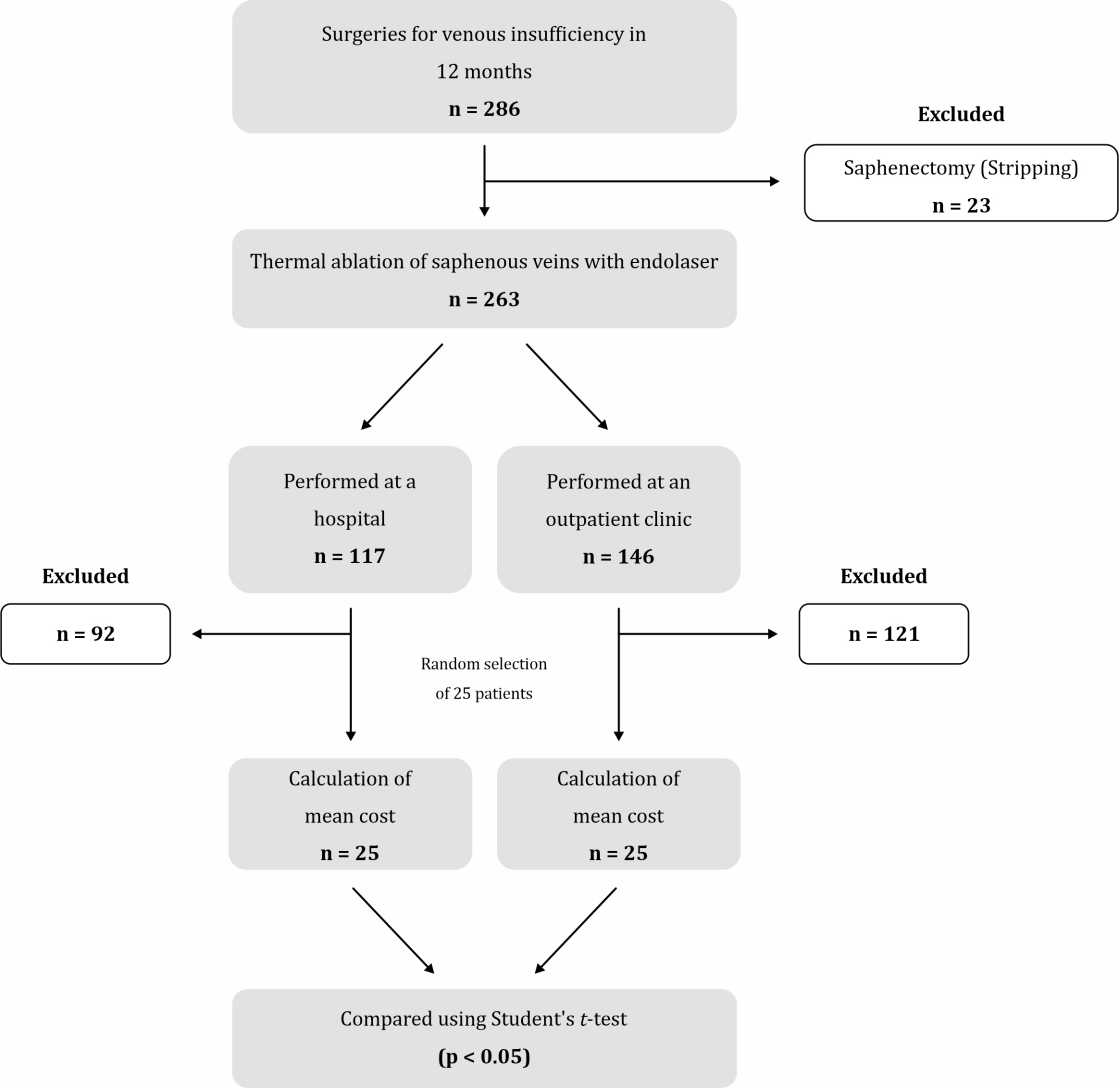
Study design diagram.

The inclusion criterion was endolaser surgery, and the exclusion criterion was saphenectomy (stripping). In total, 25 procedures were performed at a tertiary hospital and 25 were performed at an outpatient clinic equipped to perform procedures with local anesthesia and conscious sedation, meeting all regional and national health standards (Figure 1). The study design is outlined in Figure 2.

Although the ideal sample was calculated at n = 32 in each group, due to restrictions imposed by the *Lei Geral de Proteção de Dados* (General Data Protection Law), the operating costs of only 25 patients per group could be obtained. Patients were selected through the hospital’s electronic medical records based on the medical record number, which hid their identity. System filters were used to identify patients who underwent the procedures in the clinic or the hospital, and 25 individuals were randomly selected from each group.

To assess the procedures’ safety in hospital and clinical settings, patient records were retrospectively investigated for intra- or postoperative complications. The cost of the inpatient procedure for each patient was provided by a health insurance company, excluding physician fees, i.e., only the hospital cost was provided. The cost of the outpatient procedure, charged directly to the patient, was provided by the private clinic, also excluding physician fees. Neither estimate included the cost of the fiber used in the procedure, since it was identical for both groups. The cost included room rental, anesthesia, and materials (micropore tape, bandages, sutures, etc.). After collecting the data, the mean cost was determined for each treatment setting, and the results were compared statistically using Student's *t*-test. P-values < 0.05 were considered statistically significant. The CHEERS guidelines were followed throughout the development of this study.^11^

The study protocol was approved by the Universidade de Passo Fundo Ethics Committee (certificate 52368120.7.0000.5342; opinion 5,065,505). All patients provided written informed consent.

## RESULTS

Between January 2023 and February 2024, the medical team performed 286 procedures to treat varicose veins, 263 of which were laser thermoablation of saphenous veins. Of these, 117 were performed at the hospital and 146 at the private clinic, and 25 from each group were randomly selected. The mean total cost of the procedure (room fee and material cost, without physician fees), was BRL 1,391.99 (SD, 280.8) at the hospital and BRL 1,593.40 (SD, 99.53) at the clinic, which was a significant difference according to Student's *t*-test (p = 0.02).

Of the 25 patients who underwent in-hospital treatment, 18 were women and seven were men. Of the 25 outpatients, 20 were women and five were men. In both groups, 15 patients underwent bilateral procedures and underwent 10 unilateral procedures. No intra- or postoperative complications occurred.

## DISCUSSION

The ETA method uses heat to occlude the veins. In other words, it induces collagen shrinkage followed by fibrotic sealing of the vessel lumen.^12^ Thermal ablation of the great saphenous vein is the focus of the technique, which can be performed in an outpatient setting under local anesthesia. Residual varicosities can be treated either by sclerotherapy or by multiple phlebectomies at the beginning of treatment.^7^ Due to these factors, the number of ETA procedures in outpatient settings is gradually increasing, and the procedure has been shown to be safe and effective for varicose vein treatment,^6^ with success rates of up to 92%.^13^ The 2023 Brazilian Society of Angiology and Vascular Surgery guidelines on chronic venous disease recommend thermal ablation without ligating the saphenofemoral junction to treat great and small saphenous vein insufficiency, with a strong level of evidence (A) and a Class I recommendation.^13^

Outpatient surgery using minimally invasive procedures is becoming the method of choice among a significant portion of the medical community. In addition, many patients and family members prefer outpatient surgery due to the infrastructure and familiarity with the health care team, producing a relationship of greater trust and ease between the parties.^8^ The possibility of personalizing patient care in a private clinic is another advantage to consider. This is in line with the philosophy of outpatient surgery, in which trusted hands manage treatment and promote patient-centered, integrated, and interdisciplinary medicine.^8^

In addition to patient benefits, outpatient procedures also benefit the healthcare system.^8^ The decentralization of care alleviates overload in hospitals by reducing the number of less complex cases, allowing them to focus on surgeries and procedures that require a hospital environment.

A 2018 prospective cohort study by Varetto et al. compared ETA performed in hospital and outpatient settings, finding no significant differences in clinical success or perioperative complications. However, patients over 65 years of age preferred the outpatient setting, probably because it involved less emotional stress.^6^ Another important advantage of outpatient surgery is the reduced risk of surgical site infections. Nosocomial infections occur in 25% of hospital-based procedures^14,15^ but in only 3% of outpatient procedures.^10^

Careful selection of outpatient candidates is necessary.^8^ In individuals with higher levels of anxiety and severe varicose vein disease, sedation and spinal anesthesia may facilitate the procedure. Because anesthesiologists are available in the hospital setting, deeper sedation can be performed, which relieves anxiety, and spinal anesthesia can be used in cases of multiple varicose veins, where tumescence alone would be insufficient to eliminate the patient's pain. In individuals with a higher risk of intraoperative complications, it may be more prudent to perform the procedure in a hospital setting.^8^

This study raised a number of important points. First, safety was equivalent in outpatient and inpatient surgery, since no complications were observed in either group.

Second, although the cost difference between the treatment settings was statistically significant, it was quantitatively small, possibly with little budgetary impact. It is important to note that fiber was excluded from the cost analysis. In some centers, when the material is added to the hospital cost, the cost difference may favor outpatient surgery. More thorough cost studies are needed to clarify this issue. This factor may explain the different results of European studies, in which the cost of outpatient procedures was lower than inpatient procedures.^6,8-10^

In addition to the cost of fiber, the cause of this discrepancy requires further investigation. Input costs are another hypothesis: when negotiated in large quantities through contracts, as is done by large hospital chains, lower prices are possible. In the present study, the hospital where the procedures were performed is philanthropic, resulting in lower operating costs that can be passed on to patients.

Guillaumon and Rocha^16^ conducted a cost analysis study of outpatient and inpatient saphenectomy procedures performed at a university hospital, finding that the outpatient procedure was a better value, unlike our findings. However, this study was published in 2003, with the data having been collected between 1992 and 1998, a period far removed from the current economy. Furthermore, it is worth noting that the present study analyzed saphenous vein treatment using endolaser, and not stripping, which may also explain the divergent results.

Although the ideal sample was calculated at 32 participants per group, legal restrictions prevented us from obtaining data directly from the hospital via the health insurance company, limiting the sample to 25 patients. This limitation should be considered when interpreting the results, since the reduced sample size could have affected the study power. Regarding study randomization, this is a simple and practical method in retrospective studies, in which the medical record number functions as a neutral identifier. The non-participation of the researchers in selection process strengthened its impartiality.^17^

More robust studies with larger sample sizes conducted at several vascular health centers throughout the country are needed to assess the increase in ETA in Brazil. Therefore, understanding how the procedure is organized throughout Brazil is of utmost importance for vascular surgeons who seek to provide the best treatment for their patients.

To the best of our knowledge, this is the first study to compare inpatient and outpatient ETA in Brazil, although it has some limitations. It was a single-center study from one region of the country and included few patients. Furthermore, as a retrospective study, the analysis was limited regarding the materials, as well as the surgery time and complexity.

## CONCLUSIONS

Although the mean cost of the outpatient procedure was higher than the inpatient procedure, the advantages of outpatient surgery provide a favorable cost-benefit ratio for patients, and outpatient ETA is safe. However, further studies with larger samples conducted at different centers throughout Brazil are needed, in addition to analyses of why outpatient costs are higher than hospital costs.

## References

[1] Chung JH, Heo S (2024). Varicose veins and the diagnosis of chronic venous disease in the lower extremities. J Chest Surg..

[2] Gawas M, Bains A, Janghu S, Kamat P, Chawla P (2022). A comprehensive review on varicose veins: preventive measures and different treatments. J Am Nutr Assoc..

[3] Raetz J, Wilson M, Collins K (2019). Varicose veins: diagnosis and treatment. Am Fam Physician.

[4] Sociedade Brasileira de Angiologia e de Cirurgia Vascular (2024). Estimativas.

[5] De Maeseneer MG, Kakkos SK, Aherne T (2022). European Society for Vascular Surgery (ESVS) 2022 clinical practice guidelines on the management of chronic venous disease of the lower limbs. Eur J Vasc Endovasc Surg.

[6] Varetto G, Gibello L, Frola E (2018). Day surgery versus outpatient setting for endovenous laser ablation treatment: a prospective cohort study. Int J Surg.

[7] Darwood RJ, Theivacumar N, Dellagrammaticas D, Mavor AI, Gough MJ (2008). Randomized clinical trial comparing endovenous laser ablation with surgery for the treatment of primary great saphenous varicose veins. Br J Surg.

[8] Deindl C, Neumann A (2022). The future of outpatient surgery. Urologie..

[9] Bellani ML (2008). Psychological aspects in day-case surgery. Int J Surg.

[10] Keo HH, Spinedi L, Staub D (2019). Safety and efficacy of outpatient endovenous laser ablation in patients 75 years and older: a propensity score-matched analysis. Swiss Med Wkly.

[11] Husereau D, Drummond M, Augustovski F (2022). Consolidated Health Economic Evaluation Reporting Standards 2022 (CHEERS 2022) statement: updated reporting guidance for health economic evaluations. Value Health.

[12] Galanopoulos G, Lambidis C (2012). Minimally invasive treatment of varicose veins: endovenous laser ablation (EVLA). Int J Surg.

[13] Kikuchi R, Nhuch C, Drummond DAB (2023). Brazilian guidelines on chronic venous disease of the Brazilian Society of Angiology and Vascular Surgery. J Vasc Bras.

[14] Sewonou A, Rioux C, Golliot F (2002). Incidence of surgical site infection in ambulatory surgery: results of the INCISCO surveillance network in 1999-2000. Ann Chir.

[15] Owens PL, Barrett ML, Raetzman S, Maggard-Gibbons M, Steiner CA (2014). Surgical site infections following ambulatory surgery procedures. JAMA.

[16] Guillaumon AT, Rocha EF (2004). Análise de custos de safenectomia ambulatorial em hospital universitário. Rev Col Bras Cir.

[17] Miola AC, Espósito ACC, Miot HA (2024). Técnicas de randomização e alocação para estudos clínicos. J Vasc Bras.

