# Supplementary material for: Outpatient treatment of varicose veins with endolaser in clinic vs. hospital: cost-benefit and safety assessment
Source: J Vasc Bras. 2025 Jun 30;24:e20240160. doi: 10.1590/1677-5449.202401602 (PMC12212346; doi:10.1590/1677-5449.202401602)
Supplement: Translation

# Translation: Tratamento ambulatorial de varizes com *endolaser* em clínica × hospital: avaliação de custo-benefício e segurança

**DOI:** 10.1590/1677-5449.202401601

**Authors:** Camila Biedler Giordani, Mateus Picada Correa, Luiza Brum Borges, Vitória Cerbaro Farias, Renan Camargo Puton, Jaber Nashat Saleh, Rafael Stevan Noel, Julio Cesar Bajerski

**Affiliations:** 1 Universidade de Passo Fundo – UPF, Passo Fundo, RS, Brasil.; 2 Instituto Vascular de Passo Fundo – Invasc, Passo Fundo, RS, Brasil.

**Keywords:** cirurgia ambulatorial, termoablação endovenosa, varizes, custo-benefício

## Abstract

**Contexto:**

A doença varicosa é comum entre a população, com incidência de cerca de 38%. No Brasil, ela atinge 45% das mulheres e 30% dos homens. Atualmente, a termoablação endovenosa (TAEV) de safenas é o método de escolha no tratamento de varizes. Por ser um procedimento minimamente invasivo, que promove rápida recuperação e não exige internação hospitalar, ele pode ser realizado a nível ambulatorial.

**Objetivos:**

Avaliar a segurança e o custo-benefício do tratamento ambulatorial de varizes em clínica, comparando ao mesmo procedimento realizado em ambiente hospitalar.

**Métodos:**

Estudo transversal, retrospectivo e não controlado, do tipo série de casos, que avaliou um grupo aleatório de 50 pacientes submetidos à TAEV para tratamento de doença varicosa, realizado por um mesmo grupo de cirurgiões vasculares. Entre os pacientes, 25 realizaram o tratamento em hospital terciário e 25 ambulatorialmente. As médias dos custos de taxa de sala e de materiais dos dois grupos foram analisadas e comparadas estatisticamente com o teste *t* de Student. Complicações intra e pós-operatórias foram avaliadas.

**Resultados:**

A média do custo hospitalar do procedimento foi de R$ 1.391,99 (± 280,8) no hospital e de R$ 1.593,40 (± 99,53) na clínica. O teste *t* de Student apresentou diferença significativa, com p = 0,02. Nenhum paciente teve complicações intra ou pós-operatórias.

**Conclusões:**

A cirurgia ambulatorial, apesar de ter um custo estatisticamente maior que a hospitalar no Brasil, é segura e tem custo-benefício positivo para o paciente.

## INTRODUÇÃO

Varizes são vasos dilatados, tortuosos, palpáveis, maiores que 3 mm, e que geralmente estão localizadas nos membros inferiores^1-3^. A doença varicosa é uma das doenças mais comuns na população, com prevalência mundial variando entre 29,5 e 39,0% nas mulheres e entre 10,4 e 23,0% nos homens^2,3^. No Brasil, essa estimativa é similar. Segundo a Sociedade Brasileira de Angiologia e de Cirurgia Vascular (SBACV), 38% dos brasileiros possuem varizes (45% das mulheres e 30% dos homens) e, em pessoas com mais de 70 anos, a prevalência pode chegar a 70%^4^.

A termoablação endovenosa (TAEV) de safenas vem progressivamente substituindo a cirurgia convencional no tratamento do refluxo safênico, com recomendações fortes nas diretrizes^5^. Esse procedimento é realizado através de um acesso percutâneo sob anestesia local tumescente guiada por ultrassom^6^. Isso permite um procedimento mais preciso e uma recuperação mais rápida, diminuindo o tempo de internação pós-operatório e possibilitando que o procedimento seja também realizado a nível ambulatorial^6^.

A TAEV promove melhores resultados com menores taxas de complicações se comparada à cirurgia tradicional^3,6^, principalmente pelo fato de permitir recuperação rápida, menos dor e retorno precoce às atividades do cotidiano^7^. Na Europa, o custo-benefício de realizar o procedimento fora do hospital é positivo, porém a realidade brasileira ainda é desconhecida^8-10^. Portanto, este estudo tem como objetivo avaliar a segurança e o custo-benefício do tratamento ambulatorial de varizes no Brasil, em comparação ao mesmo procedimento realizado em ambiente hospitalar.

## METODOLOGIA

Este é um estudo transversal, retrospectivo e não controlado, do tipo série de casos. Foram selecionados aleatoriamente 50 pacientes, com base no número de prontuário, por uma pessoa que não participou do estudo. Todos os pacientes haviam sido submetidos à termoablação de veias safenas e flebectomias de tributárias, realizadas por uma equipe com 15 anos de experiência no tratamento da insuficiência venosa.

O critério de inclusão foi pacientes que realizaram a cirurgia com *endolaser*, e o critério de exclusão foi pacientes que realizaram a safenectomia (*stripping*). Ao todo, 25 procedimentos foram realizados em hospital terciário e 25 em um consultório nível 2, totalmente equipado para realizar procedimentos com anestesia local e sedação consciente, atendendo a todas as normas de vigilância sanitária vigentes no país e na região (Figura 1). O desenho do estudo está esquematizado na Figura 2.

**Figura 1:** Consultório onde as cirurgias ambulatoriais foram realizadas na clínica privada.

**Figura 2:** Esquema representando o desenho do estudo.

A amostra ideal havia sido previamente calculada em n = 32 para cada grupo. No entanto, devido às restrições impostas pela Lei Geral de Proteção de Dados (LGPD), foi possível obter apenas os custos operacionais de 25 pacientes por grupo. Assim, o estudo foi realizado com 25 pacientes em cada grupo. A seleção foi realizada por meio da plataforma hospitalar, com base no número do prontuário do sistema, sem qualquer possibilidade de identificação dos pacientes, inclusive em relação ao sexo. O responsável pela seleção apenas aplicou filtros na plataforma para identificar os pacientes que realizaram os procedimentos em clínica ou hospital, selecionando aleatoriamente 25 indivíduos de cada grupo.

Para avaliar a segurança dos procedimentos no hospital e na clínica, prontuários dos pacientes foram avaliados retrospectivamente em busca de possíveis complicações intra ou pós-operatórias. O valor do procedimento hospitalar de cada paciente foi fornecido por uma empresa de convênios médicos, excluindo-se honorários médicos. Isto é, foi fornecido somente o custo hospitalar. Já o valor do procedimento ambulatorial, cobrado diretamente do paciente, foi fornecido pela clínica privada, também excluindo-se os honorários médicos. Em nenhum deles, foi incluído o valor da fibra, já que o valor é igual para os dois grupos. O valor incluía os custos de aluguel de sala, anestesia e materiais (Micropore, bandagens, suturas, entre outros). Após a coleta dos valores, foi avaliada a média dos custos entre os dois serviços de saúde, e os resultados foram comparados estaticamente utilizando o teste *t* de Student. Foi utilizado p < 0,05 como parâmetro de diferença estatística significativa. Foram seguidas as diretrizes CHEERS para o desenvolvimento deste estudo^11^.

O protocolo foi aprovado pelo Comitê de Ética da Universidade de Passo Fundo, sob o Certificado de Apresentação de Apreciação Ética: 52368120.7.0000.5342 e parecer consubstanciado de número 5.065.505. Todos os pacientes assinaram o Termo de Consentimento Livre e Esclarecido.

## RESULTADOS

Entre janeiro de 2023 e fevereiro de 2024, dos 286 procedimentos para tratamento de doença varicosa realizados pela equipe médica, 263 foram através da termoablação de safenas com *laser*. Destes, 117 foram realizados no hospital e 146 na clínica privada. Foram selecionados aleatoriamente 25 pacientes dos 117 que realizaram o tratamento no hospital, e 25 entre os 146 que realizaram na clínica. A média do custo total do procedimento, sem os honorários médicos, foi de R$ 1.391,99 (± 280,8) no hospital e de R$ 1.593,40 (± 99,53) na clínica. Nesse custo, inclui-se o valor da taxa de sala e os custos dos materiais. O teste *t* de Student apresentou diferença significativa, com p < 0,05 (p = 0,02).

Dos 25 pacientes que realizaram tratamento intra-hospitalar, 18 eram do sexo feminino e sete do masculino. Já ambulatorialmente, foram 20 mulheres e cinco homens. Entre os 25 que realizaram no hospital, 15 fizeram procedimento bilateral e 10 unilateral. A mesma proporção foi verificada entre os pacientes da clínica. Nenhum dos pacientes teve complicações intra ou pós-operatórias. Todos os procedimentos foram realizados sem intercorrências.

## DISCUSSÃO

O método de TAEV utiliza o calor para ocluir as veias. Ou seja, induz encolhimento do colágeno seguido de selamento fibrótico do lúmen do vaso^12^. A ablação térmica da veia safena magna (VSM) é o foco da técnica, que pode ser realizada em ambiente ambulatorial e com uso de anestesia local. As varicosidades residuais podem ser tratadas tanto por escleroterapia como por múltiplas flebectomias no início do tratamento^7^. Devido a esses fatores, o número de procedimentos de TAEV em ambiente ambulatorial está aumentando gradativamente e tem-se mostrado seguro e eficaz para o tratamento da doença varicosa^6^, com taxas de sucesso de até 92%^13^. A diretriz brasileira de 2023 da SBACV sobre doença venosa crônica recomenda a termoablação sem ligadura da junção safeno-femoral para tratar a insuficiência da VSM e da veia safena parva, com forte nível de evidência (A) e alta recomendação (classe I)^13^.

A cirurgia ambulatorial, com procedimentos minimamente invasivos, está tornando-se o método de escolha entre uma significativa parcela da comunidade médica. Além disso, muitos pacientes e familiares justificam sua preferência pela cirurgia ambulatorial por conta da infraestrutura e da familiaridade com a equipe que os atende, o que gera uma relação de maior confiança e tranquilidade entre as partes^8^. A possibilidade de personalizar o atendimento do paciente em uma clínica privada é outra vantagem a ser considerada. Isso vai de acordo com a filosofia da cirurgia ambulatorial, que é de gerenciar o tratamento em mãos confiáveis, além de promover uma medicina centrada no paciente, integrada e interdisciplinar^8^.

Além dos benefícios para o paciente, o procedimento ambulatorial também favorece o sistema de saúde^8^. Graças à descentralização do cuidado, o ambiente hospitalar torna-se menos sobrecarregado com casos de menor complexidade, podendo concentrar-se em cirurgias e procedimentos que precisam necessariamente ser realizados em ambiente hospitalar.

Um estudo de coorte prospectivo, publicado em 2018 por Varetto et al., comparou a TAEV realizada em ambiente hospitalar e em ambiente ambulatorial, não encontrando diferenças entre as duas em termos de sucesso clínico e complicações perioperatórias. Entretanto, observou-se que os pacientes com mais de 65 anos preferiam realizar o procedimento em âmbito ambulatorial, provavelmente devido às menores taxas de estresse emocional promovidas por esse ambiente^6^. Outra vantagem importante da cirurgia em ambiente ambulatorial é o risco reduzido de infecções de sítio cirúrgico. Cirurgias em hospitais são responsáveis por 25% de infecções nosocomiais nos pacientes^14,15^, enquanto as ambulatoriais representam apenas 3%^10^.

Vale ressaltar que é importante selecionar o melhor paciente para a realização do procedimento em âmbito ambulatorial^8^. Em indivíduos com níveis mais elevados de ansiedade e doença varicosa bem exacerbada, a sedação e a raquianestesia podem beneficiar o bom andamento do procedimento. Devido à presença do médico anestesista em ambiente hospitalar, é possível realizar uma sedação mais profunda, aliviando o grau de ansiedade, assim como utilizar a raquianestesia em casos de múltiplas varizes, nos quais somente a tumescência não seria o suficiente para eliminar a dor do paciente. Já em indivíduos com maiores riscos de complicações intraoperatórias, a realização do procedimento em ambiente hospitalar pode ser uma alternativa mais prudente^8^.

Este estudo apresentou pontos importantes que devem ser levados em consideração. Primeiro, em relação à segurança, o estudo demonstrou que a cirurgia ambulatorial e a hospitalar apresentaram igualdade de segurança, visto que não houve complicações em nenhum dos grupos.

Segundo, apesar de haver diferença estatística de custos entre os dois ambientes, essa diferença foi quantitativamente baixa, possivelmente sem impactos orçamentários significativos. É importante salientar que a fibra foi excluída da análise de custos. Em alguns centros, onde o material é adicionado ao custo hospitalar por ser fornecido pelo hospital, a diferença de custos pode favorecer a cirurgia ambulatorial. Estudos que considerem essa disposição de custos devem ser realizados para esclarecer a questão. Esse fator pode justificar a diferença de resultados em relação a estudos europeus, nos quais os procedimentos ambulatoriais apresentaram custos menores que os hospitalares^6,8-10^.

A causa dessa discrepância, além do custo da fibra, ainda precisa de uma investigação mais aprofundada. Os custos dos insumos representam outra hipótese. Quando negociados em grandes quantidades e por contratos, como é feito pelas grandes redes hospitalares, os preços podem ficar mais atrativos. No caso do presente estudo, o hospital onde os procedimentos foram realizados é filantrópico, o que implica um custo operacional menor, podendo repassar um preço mais competitivo ao paciente.

Guillaumon e Rocha^16^ realizaram um estudo de análise de custos de safenectomia ambulatorial, comparando-a ao mesmo procedimento em bloco cirúrgico, ambas em um hospital universitário, e observaram um valor mais atrativo para a cirurgia ambulatorial, ao contrário do encontrado no presente estudo. Entretanto, o estudo foi publicado em 2003, com dados coletados entre 1992 e 1998, refletindo uma realidade econômica totalmente diferente da atual. Além disso, vale ressaltar que o presente estudo fez uma análise de tratamento de safenas por *endolaser*, e não por *stripping*, fato que também pode justificar os resultados divergentes.

Apesar da amostra ideal ter sido calculada em 32 participantes por grupo, a aplicação da LGPD impôs limitações quanto ao acesso às informações dos pacientes. Essa restrição dificultou a obtenção dos dados diretamente do hospital via empresa de convênios, limitando a amostra aos 25 pacientes cujo custo operacional pôde ser disponibilizado. Essa limitação deve ser considerada na interpretação dos resultados, uma vez que um tamanho amostral menor pode impactar o poder estatístico do estudo. Já em relação à randomização do estudo, trata-se de um método simples e prático em estudos retrospectivos, no qual o número de prontuário funciona como identificador neutro. A não participação do pesquisador nessa escolha fortalece a imparcialidade do processo^17^.

Estudos mais robustos, com amostragens maiores e que avaliem diversos centros de saúde vascular no país são necessários devido a esse aumento crescente de TAEV no Brasil. Por isso, entender como o procedimento se organiza em todo o território brasileiro é de suma importância para os cirurgiões vasculares que buscam proporcionar o melhor tratamento aos seus pacientes.

Até onde se sabe, este estudo é o primeiro a comparar a TAEV realizada em clínica e hospital no Brasil. Todavia, apresenta algumas limitações. É um estudo de centro único, com poucos pacientes, ilustrando a realidade de apenas uma região do país. Além disso, por ser retrospectivo, há limitações nas análises referentes aos insumos utilizados, ao tempo e à complexidade cirúrgica.

## CONCLUSÃO

Apesar de o procedimento realizado na clínica ter uma média de custo superior ao procedimento em hospital, as vantagens que a cirurgia ambulatorial promove ao paciente conferem uma relação custo-benefício favorável. Além disso, a TAEV realizada fora do ambiente hospitalar é segura. No entanto, são necessários mais estudos, contendo uma amostragem maior, envolvendo diferentes centros e realidades de um país continental, além de análises sobre os motivos pelos quais o custo ambulatorial no Brasil é maior que o hospitalar.
